# Doping graphene films via chemically mediated charge transfer

**DOI:** 10.1186/1556-276X-6-111

**Published:** 2011-01-31

**Authors:** Ryousuke Ishikawa, Masashi Bando, Yoshitaka Morimoto, Adarsh Sandhu

**Affiliations:** 1 Department of Electrical and Electronic Engineering, Tokyo Institute of Technology, 2-12-1 O-okayama, Meguro, Tokyo 152-8552, Japan; 2 G-COE Program on Evolving Education and Research Center for Spatio-Temporal Biological Network, 4259 Nagatsuta Midori-ku, Yokohama 226-8501, Japan; 3 Electronics-Inspired Interdisciplinary Research Institute (EIIRIS), Toyohashi University of Technology, 1-1 Hibarigaoka, Tempaku-cho, Toyohashi, Aichi 441-8580, Japan

## Abstract

Transparent conductive films (TCFs) are critical components of a myriad of technologies including flat panel displays, light-emitting diodes, and solar cells. Graphene-based TCFs have attracted a lot of attention because of their high electrical conductivity, transparency, and low cost. Carrier doping of graphene would potentially improve the properties of graphene-based TCFs for practical industrial applications. However, controlling the carrier type and concentration of dopants in graphene films is challenging, especially for the synthesis of *p*-type films. In this article, a new method for doping graphene using the conjugated organic molecule, tetracyanoquinodimethane (TCNQ), is described. Notably, TCNQ is well known as a powerful electron accepter and is expected to favor electron transfer from graphene into TCNQ molecules, thereby leading to *p*-type doping of graphene films. Small amounts of TCNQ drastically improved the resistivity without degradation of optical transparency. Our carrier doping method based on charge transfer has a huge potential for graphene-based TCFs.

## Introduction

Transparent conductive films (TCFs) are a class of extremely important components of modern technology for applications such as optical devices and solar energy utilization [1]. Indium tin oxide (ITO) is the most widely used material as TCFs; however, the high cost and the limited supply of indium, a rare-earth metal, have become a serious concern. Thus, alternative materials with high transparency and low electrical sheet resistance comparable to ITO are required. During the last decade, a number of materials, such as conducting polymer films [2] or nanostructured thin films [3] have been proposed as alternatives to ITO. Recently, carbon nanotubes have also shown high potential as the replacement material of ITO; however, their cost performance remains an issue [4].

Meanwhile, graphene, a single atomic layer of carbon, has attracted greater attention as an alternative material of TCFs because of its high electrical conductivity and transparency [5]. In addition to its superb properties, graphene-based TCFs could also be cost-competitive if produced via a chemical production method. Therefore, we focused on developing an inexpensive chemical fabrication procedure in liquid phase without any vacuum systems.

The problem of high resistivity of chemically derived graphene-based TCFs [6] still remains to be resolved. Up to now, several types of carrier doping of graphene have been demonstrated including boron- or nitrogen-substitutional doping [7,8], deposition of alkali metal atoms [9], adsorption of gaseous NO_2 _[10], and charge transfer from conjugated organic molecules [11,12]. However, controlling the carrier type and concentration of dopants in graphene films is challenging, especially for fabrication of *p*-type films. With a view to improving the electrical properties of graphene-based TCFs, we propose a novel carrier doping method based on charge transfer from conjugated organic molecules. It is anticipated that liquid phase chemical interaction between graphene and conjugated organic molecules induces a high doping efficiency.

**Figure 1:**
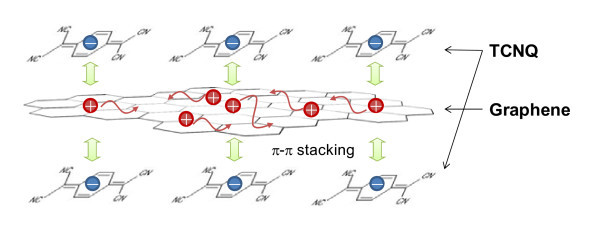
**Schematic image of doping graphene by adsorbed TCNQ molecules**.

Tetracyanoquinodimethane (TCNQ) is well known as a powerful electron accepter and is expected to favor electron transfer from graphene into TCNQ molecules, thereby leading to *p*-type doping of graphene films. Figure 1 shows a schematic image of graphene doping by adsorbed TCNQ molecules. In fact, small amounts of TCNQ improved the resistivity by two orders of magnitude without degradation of optical transparency. Our new doping method opens up the possibility of graphene-based TCFs.

## Experiment

### Synthesis of graphene

Chemically derived graphene was synthesized by the modified Hummer's method [13], a well-known approach to produce monolayered graphene via liquid-phase exfoliation of graphite oxide. Natural graphite powder (SEC Carbon SNO-30) was washed in H_2_SO_4 _and K_2_S_2_O_8_, and oxidized in KMnO_4 _and H_2_SO_4_. After centrifugation, the resulting graphite oxide was exfoliated into graphene oxide (GO) by ultra-sonication (100 W, 30 min, 60°C). Then, a GO aqueous dispersion was produced by centrifugation and dialysis to neutralize a pH. The morphology of GO synthesized by this procedure was characterized by Raman spectroscopy (excited by 532-nm Ne laser) [14], optical microscope, scanning electron microscope, and atomic force microscope (in tapping mode using Si tips).

A reduction step of GO into graphene plays an essential role to determine the electrical properties of the resulting graphene films. GO was reduced as follows: GO was dispersed in aqueous solution containing N_2_H_4_, a strong reductant, with NH_3 _to adjust pH [15]. This was reacted in 95°C water bath for 1 h, and the color of dispersion changed from brownish color to gray. Finally, the solvent of reduced graphene oxide (RGO) dispersion was replaced by *N*,*N*-dimethylformamide (DMF) using an evaporator. RGO can be dispersed well in many kinds of organic solvents including DMF, while it is easily aggregated in aqueous solution because of its low electrostatic repulsion force. A RGO sample deposited on Au (10 nm)/SiO_2 _(90 nm)/Si substrate was prepared for the evaluation of the reduction state by x-ray photoelectron spectroscopy (monochrome Al Kα X-ray).

### Fabrication of graphene films

Our graphene films were deposited on glass substrates (Corning7059) by a spray-coat method at a substrate temperature of 200°C in an atmosphere containing the solvent vapor. The thickness of the films was controlled by varying the spray amounts. The optical transmittance was measured in the wavelength range from 250 to 2500 nm, and the sheet resistance was measured by van der Pauw method.

### Doping graphene films

Doping graphene via charge transfer by TCNQ molecules was carried out as follows. First, 0.01 g of TCNQ powder (>98.0%, Tokyo Chemical Industry Co. Ltd., Tokyo, Japan) was dissolved into 5 ml of DMF solvent. It is expected that TCNQ molecules in DMF are radicalized [16]. Then, 5 ml of RGO dispersion and radicalized TCNQ in DMF were mixed and stirred for 1 week at room temperature. The color of mixture solution changed from yellow-green to orange. This RGO-TCNQ mixture dispersion has been very stable for over a few months, and no clear evidence of aggregation was observed.

## Results and discussion

### Characterization of GO and graphene

**Figure 2:**
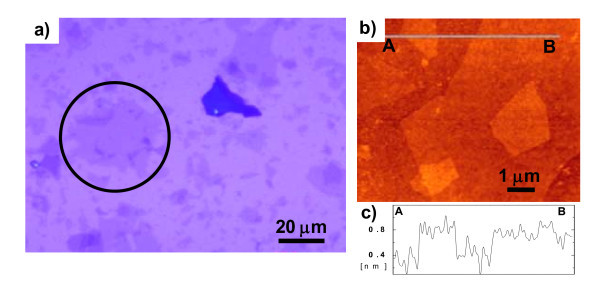
**Images of synthesized GO flakes**. **(a) **Optical microscope image of synthesized GO flakes, **(b) **AFM height image of monolayer GO flakes, and **(c) **line profile in image **(b)**.

Large GO flakes (over 30 × 30 μm^2^) were present in the GO aqueous dispersion as shown in Figure 2a. The surface morphology of these flakes was measured to be atomically thin (0.4 nm) two-dimensional (2D) structure using AFM as shown in Figure 2b,c, indicating the presence of monolayer of GO. In addition, a Raman peak shift and peak shape of second-order two phonons process peak at 2700 cm^-1^, referred to as the 2D band, which indicates about 25% of GO flakes were single layer of carbon as demonstrated in our previous article [14].

**Figure 3:**
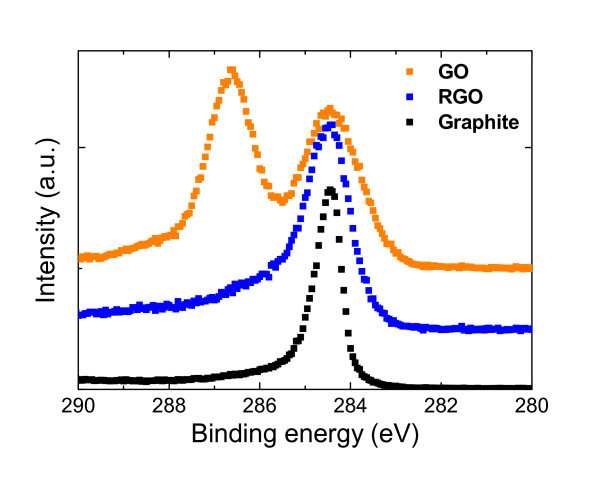
**Carbon 1s core level XPS spectra of GO, RGO, and graphite samples**.

The carbon 1s core level XPS spectra of GO, RGO, and graphite samples were shown in Figure 3. From the semi-quantitative analysis by XPS, the relative amount of oxygen containing functional groups in each sample was estimated. Peak separation was carried out for all samples after Shirley background was subtracted. The relative ratios of each component consisted of aromatic rings (284.6 eV), C-OH (286.5 eV), C-O-C (287.0 eV), and O = C-OH (288.3 eV) are summarized in Table 1. Oxygen-containing functional groups decreased from around 50 to around 25% of all components after reduction process. Such a low concentration of oxygen-containing functional groups is comparable to the RGO reduced by high-temperature annealing [17].

**Table 1:** Relative ratio of all components for each sample

Components	C-C (%)	C-OH (%)	C-O-C (%)	O = C-OH (%)
GO	49.10	25.64	22.07	3.18
RGO	73.65	19.08	0.00	7.26
Graphite	99.7	0.00	0.25	0.68

### Graphene films

**Figure 4:**
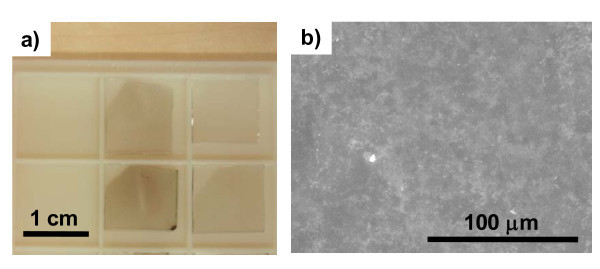
**Images of fabricated graphene films on glass substrate**. **(a) **Photograph, and **(b) **SEM image.

**Figure 5:**
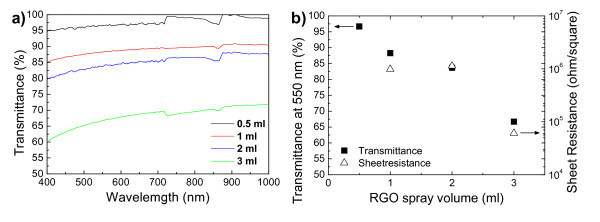
**Physical property of fabricated graphene films**. **(a) **Optical transmittance spectra, **(b) **Summarized optical and electrical properties.

Figure 4a shows photograph of fabricated graphene films on glass substrates at various spray volumes. SEM images of fabricated graphene films revealed them to be continuous and uniform (Figure 4b). Figure 5a shows the optical transmittance spectra of these fabricated graphene films, and the transmittance decreased for all wavelength ranges as the spray volume increased. Optical and electrical properties are summarized in Figure 5b. Sheet resistance of minimum spray volume sample was too high to be measured by our analyzer. The graphene films obtained in this study had a sheet resistance as high as 1 × 10^6 ^Ω/square with a transparency of 88% at 550 nm. Such a sheet resistance was the lowest obtained compared with previously reported chemically derived graphene films as deposited [6,18]. Post-annealing treatment was expected to improve the performance of our graphene films due to removal of residual solvent and oxygen-containing functional groups on RGO. Actually, Becerril et al. [19] obtained the highest performance in chemically derived graphene films through high-temperature annealing in vacuum. However, no post-annealing treatment on our graphene films was conducted, since the focus was on an inexpensive fabrication procedure without any vacuum systems.

### Doping graphene films

**Figure 6:**
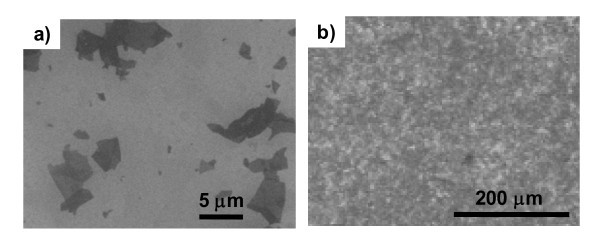
**SEM image of (a) individual doped graphene, (b) fabricated doped graphene films**.

The SEM images of individual doped graphene flakes indicate RGO flakes maintaining 2D structures after interaction with TCNQ molecules in liquid phase as shown in Figure 6a. Continuous and uniform film morphology of the doped graphene films was confirmed by SEM images as shown in Figure 6b.

**Figure 7:**
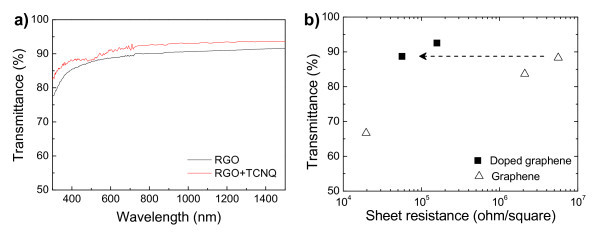
**Physical property of fabricated doped graphene films**. **(a) **Optical transmittance spectra, **(b) **Summarized optical and electrical properties.

Figure 7a shows optical transmittance spectra of doped and undoped graphene films at the same spray volumes. Except for an appearance of slight adsorption around 500 nm, spectrum did not change dominantly after doping. Transmittance (at 550 nm) as a function of sheet resistance of doped and undoped graphene films is summarized in Figure 7b. Owing to carrier doping from TCNQ, the sheet resistance drastically decreased by two orders of magnitude without degradation of optical transparency. To the best of our knowledge, such drastic doping effects have never been achieved until now [20]. However, the estimated sheet carrier concentrations were 9.96 × 10^10 ^and 1.17 × 10^12 ^cm^-2 ^for the undoped and doped graphenes, respectively. These estimated values are similar to the reported values by Coletti et al. [21]. They modified the carrier concentration of monolayer epitaxial graphene on SiC by one order of magnitude by deposition of tetrafluoro-TCNQ. In short, the better doping effect cannot be interpreted only by accelerated charge transfer induced by radicalized TCNQ molecules in DMF solvent. Further it is necessary to consider other factors such as improvement of film stacking or percolation effect.

## Conclusion

The authors developed a new and inexpensive fabrication method of chemically derived graphene-based TCFs and demonstrated a huge potential of doping effect via charge transfer by conjugated organic molecules. All of the fabrication steps including the reduction of GO and carrier doping were carried out in liquid phase. Therefore, this novel method proposed in this study does not require any vacuum system and is suitable for quantity synthesis. Furthermore, chemically derived graphene combined with the above doping technique could be a potential alternative to conventional transparent conductive materials.

## Abbreviations

DMF: *N*,*N*-dimethylformamide; GO: graphene oxide; ITO: indium tin oxide; RGO: reduced graphene oxide; TCFs: transparent conductive films; TCNQ: tetracyanoquinodimethane.

## Competing interests

The authors declare that they have no competing interests.

## Authors' contributions

RI designed and conducted all experiments and characterisation and drafted the manuscript. MB helped in technical support for experiments and drafting the manuscript. Both YM and AS have read and approved the final manuscript.
